# Contextual Cross-Referencing of Species Names for Fiddler Crabs (Genus *Uca*): An Experiment in Cyber-Taxonomy

**DOI:** 10.1371/journal.pone.0101704

**Published:** 2014-07-08

**Authors:** Michael S. Rosenberg

**Affiliations:** School of Life Sciences and Center for Evolutionary Medicine and Informatics, The Biodesign Institute, Arizona State University, Tempe, Arizona, United States of America; University of Colorado, United States of America

## Abstract

Cyber-taxonomy of name usage has focused primarily on producing authoritative lists of names or cross-linking names and data across disparate databases. A feature missing from much of this work is the recording and analysis of the context in which a name was used—context which can be critical for understanding not only what name an author used, but to which currently recognized species they actually refer. An experiment on recording contextual information associated with name usage was conducted for the fiddler crabs (genus *Uca*). Data from approximately one quarter of all publications that mention fiddler crabs, including 95% of those published prior to 1924 and 67% of those published prior to 1976, have currently been recorded in a database. Approaches and difficulties in recording and analyzing the context of name use are discussed. These results are not meant to be a full solution, rather to highlight problems which have not been previously investigated and may act as a springboard for broader approaches and discussion. Some data on the accessibility of the literature, including in particular electronic forms of publication, are also presented. The resulting data has been integrated for general browsing into the website http://www.fiddlercrab.info; the raw data and code used to construct the website is available at https://github.com/msrosenberg/fiddlercrab.info.

## Introduction

There are numerous projects focused on making literature on taxonomic names more accessible and useful [1]. Taxonomy databases such as the World Register of Marine Species (WoRMS) and the Integrated Taxonomic Information System (ITIS) are focused primarily on providing authoritative lists of names, along with synonymy. The Biodiversity Heritage Library (BHL) is digitizing and providing open access to millions of pages of taxonomic literature. Projects such as BioNames [2] attempt to link across major resources, including databases of texts, taxonomic names, and phylogenetic trees. The Global Biodiversity Information Facility (GBIF) tracks and links museum specimens with names and collection locations. Many cybertaxonomy projects have automated extraction of taxonomic names from electronically available literature at their core. The potential usefulness of these approaches and resources is huge. However, one area in which these projects explicitly fail is context. While they can discover/recognize that a specific name appears in a particular publication, they do not (and arguably, cannot) determine the context in which the name was used, and sometimes this context is extremely important for understanding both what the author meant and the currently recognized species to which they were actually referring [3], [4]. For example, an automated search of Hoffmann [5] and Kingsley [6] might discover that both publications use the species name *Gelasimus marionis* Desmarest, 1823. Cross-referencing this name against WoRMS would indicate that today this name is recognized as a junior synonym for the fiddler crab *Uca vocans* (Linnaeus, 1758). What it fails to discover is that Hoffmann was referring to a species found in Madagascar, while Kingsley was referring to a species found in the Philippines. For the last 40 years, it's been recognized that what used to be called *Uca vocans* consists of a complex of closely related species; *Uca vocans sensu stricto* is found throughout parts of the western Pacific Ocean, while the species found in the Indian Ocean (including Madagascar) to which Hoffmann refers is today known as *Uca hesperiae* Crane, 1975. Because this is not simply an issue of synonymy and priority, without understanding the context in which the name was used, it is both difficult for automated approaches to correctly identify the species that Hoffmann studied and to recognize that these two papers refer to different species as we understand them today.

In addition, most of the literature-based projects are preferentially focused (for good reasons) on taxonomic journals and papers. However, for greatest usefulness and coverage it will eventually be critical to include all literature, not just taxonomic literature, in these endeavors. The goal here is not just to resolve taxonomic uncertainty (accurately identify the correct species) in systematics, but in experimental studies as well. Without inclusion of taxonomic usage in experimental studies, we run the risk of not recognizing experimental variation due to phylogenetic variation, potentially bias systematic reviews and meta-analyses due to incorrect species designation, and generally make comparative analyses more difficult. For example, the most widely studied fiddler crab in experimental work has likely been *Uca pugilator* (Bosc, 1802) [7; personal observation], a species with a geographic range that used to be thought to include the entire Atlantic coast of the United States, including the Gulf of Mexico, from Massachusetts through Texas. Based in part on the recognition of minor color morphs with variance in physiological response to experimental conditions [8], [9], in 1974 *U. pugilator* was split into two species [10], the traditional form located on the Atlantic coast from Massachusetts through northwestern Florida, and a new species, *U. panacea* Novak and Salmon, 1974, which overlaps with *U. pugilator* in northwestern Florida but extends west to Texas. Thus, experimental studies on “*U. pugilator*” which predate the recognition of *U. panacea* (or which are unaware of the taxonomic change) may or may not be recognized as the correct species, depending on where the specimens were collected (one of the primary biological supply companies which provide fiddler crabs for experimental studies is located right at the sympatric zone, further complicating the issue). Vernberg and Costlow [11] reported metabolic differences in *U. pugilator* from Florida versus those from North Carolina and New York; the importance and interpretation of this variation changes if it turns out that the Florida specimens are a different species. Generally, taxonomists focus on prior taxonomic literature and thus tend not to revise or comment upon taxonomic names found in experimental studies.

In an effort to resolve these types of problems, I conducted an experiment in cyber-taxonomy focused on identifying the context of name use. Throughout, the term “context” is used in a similar, although slightly broader, sense to that of concept taxonomy [3], [4]. It needs to be stated up front that this effort was an experiment and not meant to serve as a general approach to solving these issues. I was not trying to invent a system that would generally solve the problem; instead, my goal was to test an approach in recording, resolving, and parsing context. This report is intended to highlight issues which have not generally been discussed in the literature in the hope that it may help guide others interested in finding better approaches and solutions to these sorts of problems.

For this study I focused on the genus *Uca*, the fiddler crabs. It is of a relatively manageable size (102 extant species are currently recognized), with extensive literature, and a history of occasionally complex systematic confusion. Prior to this project a database with approximately 2,500 known references to the genus had already been constructed, with over half of the publications already collected in either paper or electronic form, allowing a solid starting point for working from the literature. Additionally, a long-standing website on the genus (http://www.fiddlercrab.info) provides a useful, established platform (>33,000 hits over the last year) for releasing the experimental cyber-taxonomy results which make up the focus of this study.

## Materials and Methods

### Database Design

Although other contextual schemes were considered it was eventually determined that taxonomic names were primarily used in one of four contexts: (1) reference to a specimen, (2) reference to a geographic location, (3) reference to a literature citation, and (4) without context. Because many publications were not available in electronic form and because context could not clearly be computationally determined, all data was recorded manually in a spreadsheet.

**Figure 1:**
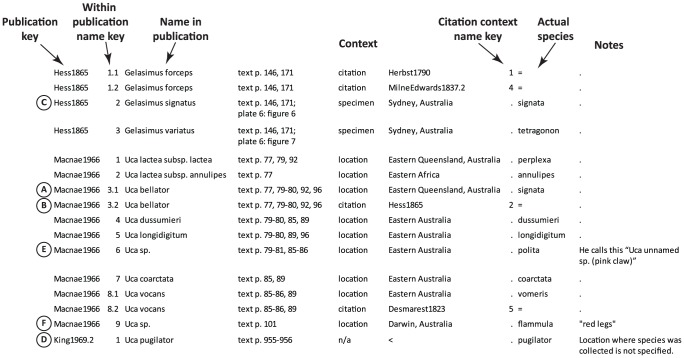
An example of contextual taxonomic name data recorded for three publications [12]–[14]. The columns represent: (1) a unique key to identify a publication; (2) a numeric key for separating different names used in a single publication and in different contexts; (3) the exact name as used in the publication; (4) where in the publication the name occurs or is applied; (5) the context of the use; (6–7) additional information on the context, with details depending on the type of context (described in text); (8) the “actual species”: either the accepted species (as we now understand it) which the authors was referring to or an equals sign (for citation contexts) indicating the accepted species should be computationally determined; (9) notes on the name usage. A period generally indicates no data (columns could not be left blank). Two additional columns of data were also recorded: the common name(s) used in the publication and notes on the publication in general. These columns were rarely used and were left out of the figure to save space. Specific records indicated with letters in circles are discussed further in the text.

For each reference to a fiddler crab appearing in a publication, the following information was recorded (Fig. 1): (1) the publication was identified with a unique key (generally a combination of author and year) which would allow cross-referencing of the publication; (2) a key to identify each unique name used in the publication—later this was expanded to allow identification (or ignore when necessary) of the distinct context of each name when it was used in multiple contexts. This was necessary since citing authors often do not apply their use of a name to the entirety of contexts in which it was used in the original publication (see below); (3) the scientific name as used in the publication, with the exact spelling and capitalization preserved; (4) when applicable, the common name associated with the scientific name. Some few publications only used common names, but were otherwise important for context or history (while sometimes interesting in their own right, common names are not the focus of this study); (5) where in the publication the scientific name occurs or is applied (e.g., page numbers, figures, plates, etc.); (6) the context of how the name was used (multiple columns of the spreadsheet, described in detail below); (7) either the correct species name as we recognize it today or an indication that the species should be determined through computational cross-referencing (see below); (8) general notes on the publication or the specific use of the name in that publication (for example, if a name was used as a type description).

Most of the records in the database contain species level names, but data on specific discussions of generic and subgeneric names (even absent of species) were also recorded, since these are both generally important and of taxonomic interest. All spelling variants, including typographical errors, were maintained in the primary records to allow and demonstrate the degree of variation found in the literature. A separate table was constructed to allow matching of spelling and typographic synonymy to the accepted spelling. For example, the species name *coarctata* has also been recorded in the literature as *coarctatus, coartatus*, and *corctata*. The first of these is a deliberate variant based on taxonomic gender-matching rules with a genus (*Gelasimus*) of alternate gender; the latter two are mistakes due to either typographical errors or confusion by authors.

#### Recording context

The specific contextual data recorded depended on the type of context. For both specimen and location contexts (which in the end were largely treated identically), the geographic location associated with the specimen/location was recorded. When available for specimen contexts (which was rare, particularly for older publications), museum lot or specimen numbers were often recorded as well. The specimen context was generally reserved for explicit taxonomic studies and museum depositions. Experimental studies which used a species as study subjects but did not otherwise keep the specimens at the completion of the experiment were recorded as locations (based on where the specimens were obtained).

For citation contexts, the cited work was recorded (based on the publication key, Fig. 1, column 1), as well as the key indicating the name in the cited work to which the citation applies (Fig. 1, column 3). This key could be recorded as either a general citation to the use of that name, or to a specific contextual use in the original work. For example, an author (of work A) might use the name *Uca pugilator* and generally cite *U. pugilator* in an earlier work (work B); in this case, one can apply the citation to all contexts of *U. pugilator* in work B. In another case, an author (of work C) might use *U. pugilator*, but specifically cite only part of a previous publication (work D) (for example by using the phrase “in part” in a taxonomic context). In this case, the citation needs to specify only the relevant cited contexts. This distinction was recorded by using a combination of integer and decimal keys. Each unique name was given a different integer base as the key (starting with 1). When multiple contexts appeared in a paper, each context was given an additional decimal code to the key (1.1, 1.2, 1.3, etc.). A citing paper could either be coded with just the integer portion (referring to all contexts with that integer base) or to the full decimal key (referring only to that specific context). When a citing paper referred to multiple, but not all, contexts, independent citation entries were made for each cited context. In rare cases, citations to a publication were general and not specific to any internal context; these were coded by reserving the key zero for such citations. When the cited publication has not been added to the name database (whether due to lack of access or because it has yet to be recorded), cross-referencing to the specific context could not be determined and a period is used as a placeholder to indicate the missing data.

In Figure 1, Macnae [12] uses *Uca bellator* (Adams and White, 1848) in two contexts: in the first (record A), he refers to a species found in Eastern Queensland, Australia; in the second (record B), he applies the name to a citation [13], specifically to name #2 found in Hess (record C): *Gelasimus signatus* Hess, 1865. If a later author applied a name to all of Macnae's uses of *U. bellator*, we would record that citation as Macnae1966 | 3 (referring to both records A and B in Figure 1). If they referred to only a specific context (e.g., Fig. 1, record A), we would record that citation as Macnae1966 | 3.1.

Many publications used taxonomic names without any specified context. There were three primary reasons for this: first, they may have stated a general reference to a species assuming any reader knew precisely to what they were referring (e.g., “One example of a species with this behavior is *Uca pugnax*.”). Without a citation or a specification of a specimen or location, these were considered without context. Second, there are some experimental studies which record a study species but otherwise do not provide any information on where the specimens were obtained. When applicable and not otherwise specified, the location of the lab of the authors was assumed to represent the collection location; in cases where this makes no sense, it was recorded as without context. For example, King and Siddall [14] report a study which includes *U. pugilator*, but without any explanation of where the specimens were obtained. Both investigators were located in the San Francisco area, but *U. pugilator* is not found on the Pacific coast of the United States (in fact, no fiddler crabs are found north of San Diego); therefore, this record had to be recorded without a specified context (Fig. 1, record D). A note is included to explain the lack of context. The third major reason for lack of context was when an author used multiple spellings for a species name in a single publication; usually one of the spellings was clearly primary with the others appearing to be typographic or editing errors. In these cases the primary spelling was used for all deliberate contextual uses by the author with the alternate spelling listed only once without context.

Data collection was focused on the use of names *considered valid by the author* of the publication. Thus names which an author treats as junior synonyms or misidentifications are not listed in the database as their own records; instead they are only included secondarily through the citation context. For example, Crane [15] mentions the name *Uca thayeri zilchi* Bott, 1954, only as a junior synonym of *Uca thayeri umbratila* Crane, 1941. The name *Uca thayeri zilchi* is not directly listed in the database as a name used by Crane; instead it is linked as one of the citation contexts of *Uca thayeri umbratila*. The exception to this rule was when an author discussed a name which they considered invalid, but which was not otherwise associated with any valid name. For example,Crane [15] discusses the name *Gelasimus huttoni* Filhol, 1886; this name was originally used to describe a species supposedly found on Campbell Island, New Zealand. The type specimen has long been lost, the description is inadequate to equate with any other species, and carcinologists do not believe fiddler crabs are found on New Zealand, making even the location of the record suspect. The name *Gelasimus huttoni* is included as a record for Crane [15], however, because there is no valid name to use as the basis of a citation record. The fact that the use of the name was considered invalid by the author is included as a note attached to the record.

### Identifying the Species

One of the key fields for each record is an estimate of the currently recognized species referred to by the author, regardless of the name actually used in their publication. There were two methods for recording this data. For citation records, no species was specified and instead a marker was used to indicate that the correct species name should be computationally determined from the cited work (see below). For all other contexts, the currently accepted name of the species was recorded. These names were consistently updated and refined as newer publications were added to the database and primary publications reexamined by later authors. In these cases, the currently accepted species name for that context was determined using a number of factors. First, high quality taxonomic works from recent authors were given priority over older works in determining the name as recognized today. Generally, the most recent taxonomic treatment was assumed to be correct unless there was clear reason to believe it to be incorrect. Second, our modern understanding of geographic distributions was used to adjust names when the recorded location strongly implied a different species than used in the original publication. For example, all references to *Uca vocans* and its junior synonyms—*Gelasimus marionis, G. cultrimanus* Adams and White, 1848, *G. nitidus* Dana, 1851, and their derivatives—found from the east coast of Africa through India are assumed to refer to *U. hesperiae* since that is the currently accepted name for the only *vocans*-group species known from those geographic regions. If no conflicting information was present, the species as originally stated by the author (or the currently recognized senior name for cases of synonymy) was assumed to refer to the same concept as in use today. In cases where the currently recognized name was ambiguous, this correct name was recorded as unknown or indeterminate (often with an accompanying explanatory note). When a single record was later known (or believed) to refer to multiple species, the record was duplicated (with each receiving a unique species id key) for each correct species. An example of this is Gould [16], where he uses the name *Gelasimus vocans* to refer to a fiddler crab from Massachusetts in the United States. It is widely believed that he was jointly referring to both *U. pugiltor* and *U. pugna*x (Smith, 1870) (and many later authors cite him for both in later taxonomic works) so both were listed as separate records even though he did not make this distinction.

The records illustrated in Figure 1 include both cases where the name used by the author matches the name used today and cases where the name does not match. In record A, the use of *Uca bellator* actually refers to the species *U. signata*; in the second use of that name, record B, the actual species needs to be determined by cross-referencing the citation (in this case, it also will turn out to apply to *U. signata*). On the other hand, the uses of *U. dussumieri* (Milne Edwards, 1852) and *U. longidigitum* (Kingsley, 1880) match our understanding and use of names today. Macnae [12] also mentions two undescribed (at that time) species. He refers to the first (record E) as “*Uca* unnamed sp. (pink claw).” This species was subsequently described by Crane [15] as *U. polita* Crane, 1975. The other unknown species he mentions (record F) is from Darwin, Australia, with “red legs” and is now considered to be *U. flammula* Crane, 1975 [17].

#### Computational identification of accepted species

While it was decided that primary contextual uses of specimens and locations needed to have the “correct” species name recorded (and updated when necessary) with the original record, citation contexts did not have this restriction and instead could have the correct species name determined computationally from the cited work. Because most uses of names are likely to be citations to other works, this drastically reduces the number of corrections which need to be made as our understanding of the taxonomy of the group evolves. However, the manner of this computation is not completely straightforward. First, recall that citations can either be contextually general (i.e., applying to all contexts of the cited work) or specific (i.e., applying to only a single context of the cited work). In the latter case, it is a simple matter to copy the accepted species name from the specific cited record. In the former, however, the multiple cited records may not all refer to a single species. The basic algorithm for determining the accepted name is as follows. Given a citation record which cites multiple contexts from a previous publication:

1. Collect all of the accepted names for each cited context
2. If all of these names are the same (only one name is accepted for all cited contexts), pass that name onto the citing record and quit. Otherwise,
3. From the collected names, find out which name(s) occur most often across all of the cited contexts
4. If a single name has a plurality of uses in the cited contexts, pass that name along to the citing record and quit. Otherwise,
5. If multiple names are tied for the plurality of uses, see if any of those names matches the name used in the citing publication. This matching has to allow for spelling variation in the citing publication. If there is a match, pass that name back to the citing record and quit. Otherwise,
6. If there is not a match between the citing name and those tied at a plurality, arbitrarily use the first name in the plurality set as the accepted name.

Importantly, for any of the results from steps 4–6, the term “in part” is automatically appended to the citing record output to make it clear that the accepted species name from the cited work does not represent all species represented in the cited records.

Records have to be processed chronologically for this algorithm to work. For example, publication A from 1950 cites publication B from 1940 which cites publication C from 1930. If we try to process publication A prior to publication B we may find that the correct species in publication B are unknown, since they have not themselves yet been determined. An earlier algorithm was developed to drive down through all citations to the most basal work (i.e., until all cited records were locations or specimens), but this turned out to lead to strange and inaccurate name labeling, particularly because very early taxonomic works only recognized one (or very few) species and tended to consist of very long lists of citations to previous works. These multiple levels of citation tended to override the narrower species context which was actually the primary meaning of the citing author. Furthermore, the current algorithm is more efficient in that it can fill in all species information in a single chronological pass, without having to continually and recursively drill down through multiple levels of citation as required by the original algorithm. Although intended for different purposes, there are conceptual similarities to the approach presented here and the synonymy ranking algorithm of Huber and Klump [18].

There is no simple method to formally test the accuracy of this algorithm, given the almost 6800 citation cross-references currently recorded in the database, each of which would ostensibly have to be individually confirmed by an expert. However, manual inspection of dozens of random records, including manually following the logic chain from citation to citation to determine how the algorithm determined a particular species designation, supports its effectiveness and general accuracy.

### Data Collection

Because literature citation would clearly be a major (and potentially the primary) context for most records, it was almost immediately clear that the best approach to tackling the literature would be chronological: start with the earliest publication and move forward by year. Thus, the first paper to be coded was Marcgrave [19], the earliest known publication to mention fiddler crabs in any form.

For every publication, every name thought to be applied to a fiddler crab was included. This includes any species, regardless of the name, thought to be a fiddler crab today, as well as any species placed in the genera recognized as fiddler crabs, predominantly *Gelasimus* (historical) and *Uca* (present), even if today they are now known to be in other genera. The reason for the latter is that there are a number of names which occur in the literature—e.g., *Uca cordatus* (Linnaeus, 1763), now known as *Ucides cordatus* (Linnaeus, 1763)—which may be confused with fiddler crabs since they were once placed in the genus now recognized as belonging to fiddler crabs. Records for an individual publication could range from a single occurrence, to dozens or hundreds of entries for larger taxonomic works. Crane's monograph on fiddler crabs [15] includes over 1400 records for 124 different names (about 20 of which are names she considered invalid, but needed to be included for proper cross-referencing and data tracking, as mentioned above).

About a dozen of the publications pre-date Linnean classification of animals in 1758, but all are either regularly referenced by early post-Linnean taxonomists or otherwise serve an important role in understanding the history of fiddler crab systematics. For example, Linnaeus [20] adopted the species name *Cancer vocans* from Rumphius [21]. Also, much of the taxonomic confusion over the accepted names for both the genus, *Uca*, and the type species, *Uca major* (Herbst, 1782) has roots in these pre-Linnean publications [22]–[25].

In addition, because this exercise was focused on name usage, it was necessary to include a handful of non-traditional “publications” which never-the-less were influential in specific taxonomic name derivations: in particular, a handful of museum labels. There are 21 instances of names appearing in “traditional” literature as references to names previously only found on museum labels. In most cases these labels were simply being referenced as junior synonyms of an established name, but in a few cases they were used as the valid name for a species, two of which, *U. monilifera* Rathbun, 1915, and *U. leptodactyla* Rathbun, 1898, are still accepted today. For example, Rathbun [26] derived the species name *U. monilifera* from an unpublished name found on a museum label by Louis Agassiz (*Eurychelus monilifer*). In contrast, Verwey [27] discusses the behavior of *U. consobrinus* Verwey, 1930; this name (now known to be a junior synonym of *U. annulipes*) had never been previously published (nor has it been used as a valid name since), but was obtained by Verwey from a museum label by J.G. De Man [15]. While the unpublished labels have no formal taxonomic priority, they were deemed necessary to properly track name usage and derivation and thus included in the database.

## Results

### Literature Summary

**Figure 2:**
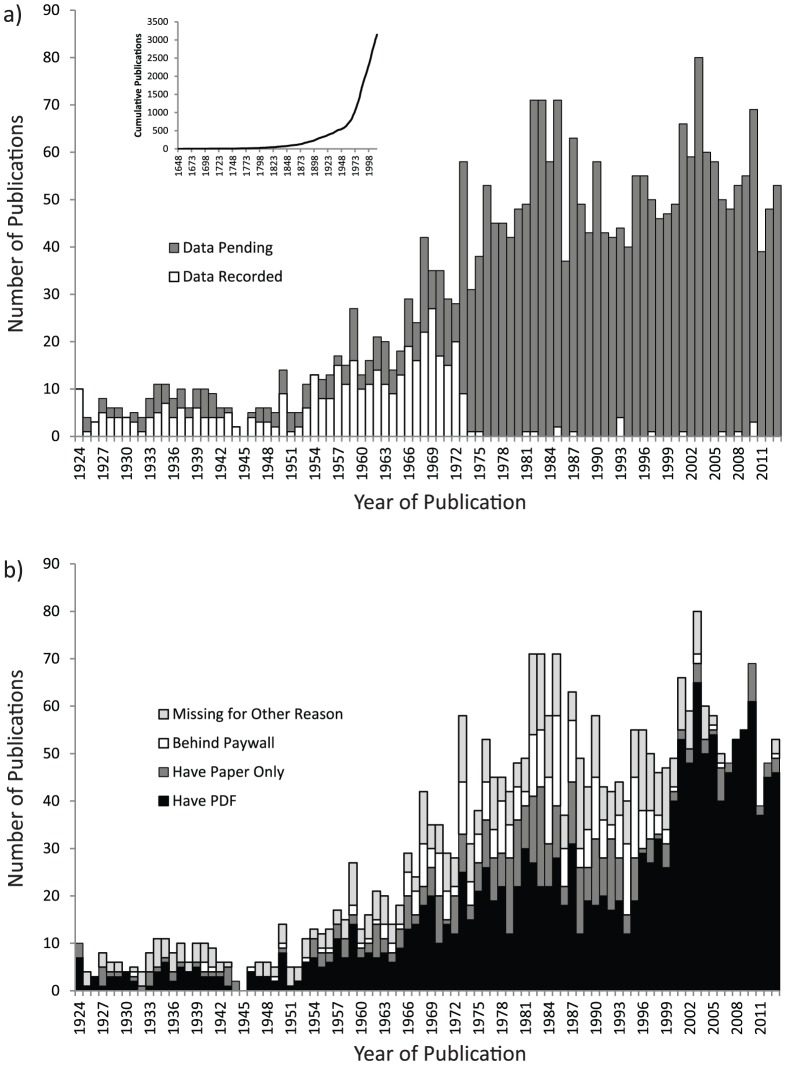
Histograms of literature illustrating pattern of publications using names associated with fiddler crabs over the past 90 years. The long tail of publications prior to 1924 are not included for clarity. a) Representation of the literature currently included in the name database versus those still pending. Approximately one quarter of all publications are in the database, including 95% of those published prior to 1924 (not shown) and two thirds of those published through 1975. The inset figure shows the cumulative number of publications in the database, by year, illustrating the explosion of literature over the past 40 years. b) Representation of the literature by accessibility. While the specific distribution is unique to my own circumstances, it generally illustrates the type of pattern one might expect to find with respect to accessibility of the primary literature.

This project would have been extremely difficult without access to the Biodiversity Heritage Library (BHL) and its excellent collection of taxonomic literature. Along with a few other electronic publication resources such as Google Books and the AToL Decapoda Literature database (http://decapods.nhm.org/references/), the BHL allowed inclusion of 95% of the pre-1924 literature in the name database, including almost every major early taxonomic work on fiddler crabs. Over 500 older publications (i.e., not newly published) were added to the fiddler crab literature database (an increase of ∼20%) simply through efforts to cross-reference citations of names as part of this project. Overall, I currently have immediate access (either a paper or electronic copy) to at least 2,329 of the 3,152 total publications known to refer to fiddler crabs (Fig. 2b). I have obtained electronic copies of 1,917 of these (either because they are open access or because of an institutional subscription); more than 460 more are available electronically but behind paywalls which I do not have access through. Overall, almost 75% of the literature is available in electronic form. Because institutional subscriptions to digitally available journals are generally focused on more recent publications, most of the inaccessible digital publications were published between 1960 and 2000.

About 64% of the publications in the literature database are from the last 40 years (Fig. 2a), only a few of which have been included in the name database at this time. Approximately two thirds of the publications through 1975 have been included in the database, with most of the missing publications being those for which I lack access (1975 was chosen as the preliminary cutoff for reporting this study because Crane's seminal monograph on fiddler crabs was published that year and it was important to make sure it was included in this experiment). A few additional papers post-1975, in particular key taxonomic works and revisions, have been preferentially added to the database to guarantee inclusion of all currently accepted names. Thus the following summaries and figures on fiddler crab taxonomy are largely based on data only up through 1975. While the remaining publications will gradually be added to the citation database, these remaining papers are largely unimportant for the purposes of reporting on the general successes and failures of this experimental approach.

### Name Summary

Ignoring spelling variation, 194 distinct specific names have been used for fiddler crabs, of which 106 are currently accepted species of fiddler crab today (102 extant and 4 extinct species). A few of the others are valid names in other genera, but most of the remaining names are junior synonyms or discarded names. Examining full binomials, multinomial names (e.g., those which include subgenus or subspecies), and spelling variants (including typos, which might not be recognized by automated scanning) there have been almost 700 combinations of names used for fiddler crab species, across 25 genera (16 genera if you ignore spelling variation in generic names). Keeping in mind that this data represents only 25% of all identified fiddler crab publications (although most of the major taxonomic works), these numbers will only increase as more data is collected. Detailed analyses of temporal and frequency patterns of name usage could be conducted on this database, answering questions about most frequently named species, most frequently misnamed species, patterns in the shift of usage from the genus *Gelasimus* to the genus *Uca*, the temporal rise and fall of taxonomic studies based on the frequency of novel names appearing in the database, etc. These sorts of analyses have not been conducted at this time because they are beyond the scope of this project, but for those who might be interested, some publishing patterns on fiddler crabs in recent scientific literature has been conducted using other resources and approaches [7].

### Data Output for Community Use

Rather than serve dynamic webpages, the entire www.fiddlercrab.info website is automatically generated from the data files on a local machine, and then uploaded to the server as an entire set. This greatly eased code and database development, as well as server load, and the system was discovered to work well as the new experimental taxonomy data was integrated into to the website. All of the data and code (Python 3 with no external dependencies) are available at https://github.com/msrosenberg/fiddlercrab.info, which contains everything necessary to recreate the entire website, except for the media files (images, photos and videos).

This new taxonomic citation data is used on the site in a variety of ways. First, there is a pair of indexes indicating all binmomial/compound and specific names (http://www.fiddlercrab.info/names). The specific name index does not include the genus and only lists the correct/accepted spelling. In contrast, the binomial/compound name index includes all variations of every name which have appeared in any publication, including names of genera, subgenera, superspecies, species, subspecies, variants, types, etc. Only variation in capitalization is ignored. Every name in each index is linked to a page with further information.

**Figure 3:**
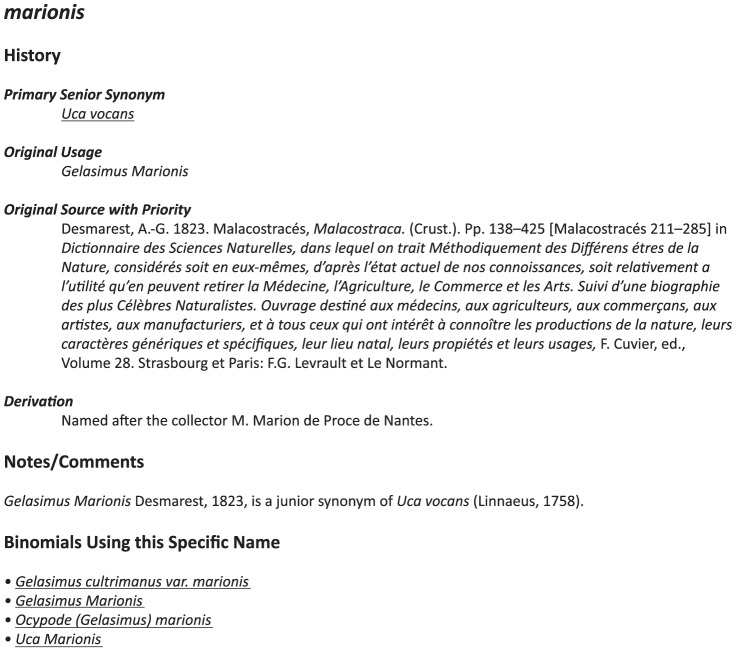
An example of the output contained on specific name pages, in this case for the name *marionis* (the full webpage can be found at http://www.fiddlercrab.info/names/sn_marionis.html). Underlined text represents links.

Each entry in the specific name index links to a page with information about that particular specific name (Fig. 3). This includes the current recognized species the name applies to (with a link to the species information page, which is independent of the specific name page); the original manner and spelling of the name; the original source with priority for that name; the etymology of the name (Latin meaning, named after a particular person, etc.) when it could be determined; and a linked list of all binomials (and other compound names) which are based on that specific name, including all spelling variants. When applicable, the page also includes notes on synonymy and homonymy; e.g., the specific name *affinis* has been involved as a homonym, having been used as a novel name twice within the genus, as well as a being a junior synonym of another name (http://www.fiddlercrab.info/sn_affinis.html).

**Figure 4:**
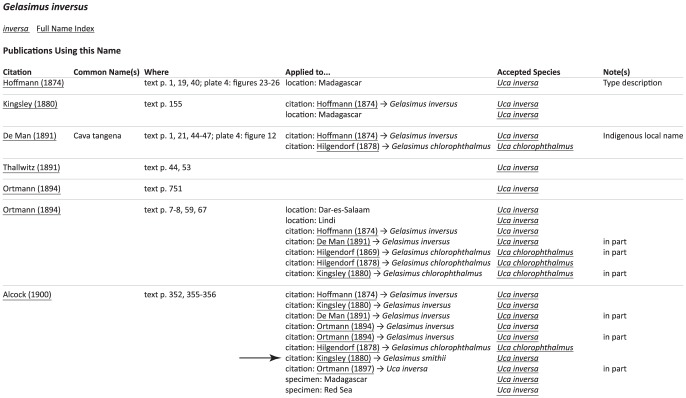
An example of the output contained on compound/binomial name pages, in this case for the name *Gelasiumus inversus* (the full webpage can be found at http://www.fiddlercrab.info/names/Gelasimus_inversus.html). Underlined text represents links. The arrow points to a particular record where Alcock (1900) applied the name *G. inversus* to Kingsley's (1880) use of *G. smithii*.

Each entry in the binomial/compound name index links to a page with information about the usage of that particular name (Fig. 4). The data displayed on these pages is essentially a cleaned up version of the data shown in Figure 1, filtered for occurrences of the particular name being examined when found in column 3 of Figure 1. Publications are listed by a traditional citation form rather than the unique key (including a link to a publication specific page); the species name key (unnecessary) and species name (redundant) are left out; and the context information is compressed into a single column, with citations including the species name in the cited paper to which the citing paper's name applies (rather than the cited paper's species key). For example, in Figure 4, one sees that Alcock (1900) applied the name *Gelasimus inversus* to Kingsley's (1880) use of the name *G. smithii*. Thus each of these pages lists every publication which has used a particular instance of a scientific name, whether it occurs only once in the literature or many times. Links on these pages include a link back to the specific name only page (described above), links to every publication mentioned on the page, and links to pages about each accepted species. In Figure 4, we can see that the name *G. inversus* has been applied to two species we recognize today: *Uca inversa* and *U. chlorophthalmus*. Since the latter is not a synonym of the former (this is a case of mistaken application/recognition of a species), a context-free analysis of the name usage would miss this relationship.

**Figure 5:**
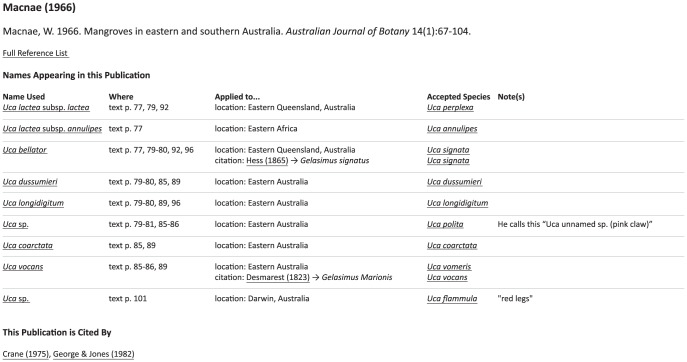
An example of the output contained on publication pages, in this case for Macnae, 1966 [12] (the full webpage can be found at http://www.fiddlercrab.info/references/Macnae1966.html). Underlined text represents links. Contrast the style and content of this output with the raw data records for the same paper illustrated in Figure 1.

Each literature reference has a unique page which is very similar in form to the binomial/compound name page just described (Fig. 5). The main difference is that the first column contains each name used in that publication (rather than listing the publication using a particular name) and the page includes a list of all papers in the database which cite the publication. Note that this is not a general citation list in the manner one would get from SCOPUS or the Web of Science. These are only citations from the name records, meaning only taxonomic name citations are included. Citations to a paper in general, without reference to a specific species, are not part of this list. In Figure 5, we see the generated output for [12] and can contrast this with the raw records for this same publication illustrated in Figure 1.

The final place where name records are used on the website are the species information pages. Pages specific to each species have been on the website since early in its development a decade ago, and includes information on the type description, synonymy, geographic range, photos, videos, etc. In addition, each species page includes a list of publications which mention that species. Historically, these references have always been based on estimates of accepted/actual species rather than simply the name found in the publication. However, this list was originally manually generated by tagging each reference (in the literature database) with every species to which it referred, as well as manually adding each citation to each applicable species page. This process did not allow for easy updating or correction of errors, nor did it efficiently explain how a particular species was actually referred to in a given publication. Given the new database, the publication list associated with a given species is now automatically generated from the data records, with a link to a page for each publication (described above) where one can find how the species is referenced. Again, these species references are based on the estimated species to which the author actually refers, rather than relying simply on the name they used in the publication. Additionally, the synonym list on each species page is auto-generated from the same data used to construct the specific name and binomial/compound name indices.

Other information and pages contained on the website (including photos, videos, and geographic ranges) have nothing directly to do with the taxonomic name database that serves as the focus of this study.

## Discussion

One of the design features of this system was to try to make it easier to update the currently recognized species referred to in older publications as our understanding of species and their distributions change. This system works easily for citation contexts, but less well for location and specimen based-contexts. For example, in 2009 we began recognizing *Uca albimana* (Kossmann, 1877) and *U. iranica* Pretzmann, 1971, as distinct species from *U. annulipes* (Milne Edwards, 1837) [28]. *U. albimana* and *U. iranica* are endemic to the Red Sea and Persian Gulf respectively, with *U. annulipes* found in the surrounding oceans. Prior to 2009 the correct name would always have been *U. annulipes.* With our new recognition, we need to identify all of the records of *U. annulipes* which should actually be one of the new species (to be precise, both of these species were proposed prior to 2009, but neither was recognized as an accepted distinct species until recently). While this can be done by finding the location associated with the context, these locations are just names, with varying degrees of specificity. Obviously searching for Red Sea or Persian Gulf is easy, but in some cases the location is to a specific city, region, or country. How does one automatically find every city or location within those regions? Currently, we cannot. Rather than associate locations with simple names, a better system might associate geographical coordinates or areas with each location/specimen and use a GIS like system that could extract every data record which overlaps with a desired area. This would allow for easier identification of records which might need to be updated as our understanding of the taxonomy changes.

Because of the design of the system, publications that cite the primary location and specimen studies do not need to be updated. Once we successfully update the base record that a specimen found in the Red Sea is actually *Uca albimana*, any publication in the database referring to that publication will have its species identity automatically updated through the cross-referencing of the system. While clearly not perfect, this at least is a general improvement on the previous system where every reference to a species (citation or primary) would have to be manually updated. Given that ∼70% of the records are citations to other works, this represents a huge decrease in potential records which need to be tracked.

The data collection for this project (which at this time has included only about 25% of known publications) is extremely tedious and it would be great to find a way to automate it. Certainly, one could imagine using automatic taxonomic-name finding methods such as those used for BioNames and similar projects to prefill much of the data. As expected, context is the difficult barrier to overcome. Even with manual inspection of a publication, it can be very difficult in many cases to figure out the context of a particular name usage. An author may mention a species and then cite a number of papers, some of which never use that species while other do. An author may list three species followed by a series of citations, each of which refers to a different combination of the three species. Sometimes specimens are referenced by a specific museum ID, other times they are more general. Many specimens are stored in lots containing multiple individuals, which can later turn out to be multiple species mixed together. Citation style and completeness has evolved through time, with older literature often using obscure abbreviations or rather loose referencing. Names of locations have changed through time and often have biased usages based on the ethnicity of the author; some of these are easy to parse, while others are quite difficult to identify today. And of course, many publications have outright errors in citation or location which confuse the entire issue even further. Because the database was constructed manually, there are undoubtedly inconsistencies in the data collected and recorded across publications.

Further complications in determining context are generated by the fact that publications occur in many languages, particularly for the older taxonomic literature. Of the 1073 publications for which I have formally determined the language so far (including every publication for which name data has been recorded), 64% are in English; German is second at 14%, with French third at 7%, and eight additional languages covering the remaining publications. Relative to the literature as a whole, these proportions are biased due to the over-representation of older literature in the publications analyzed thus far; with nearly two thirds of the known publications yet to be included, most of which are from the last 40 years, it is fully expected that the proportion of English publications will increase to over 80%. Nevertheless, there is a language barrier for much of the literature which potentially inhibits both automated and manual determination of context.

As for fiddler crabs in general, I discovered that there have been substantially more names applied to the species in the genus than one would have previously guessed. An unexpected outcome of this experiment was the discovery of a heretofore unrecognized homonymy between two species which had the potential to cause major confusion and shuffling of common usage of names if the Code of the International Commission on Zoological Nomenclature had not allowed for suppression of the senior usage [29].

Most name-centric cyber-taxonomy projects have been focused on the cataloging and cross-database-referencing of the literature on names. While not providing a generally workable solution, this study illustrates the importance, and difficulty, of dealing with taxonomic context. One hopes it will spur interest in tackling these issues as the development of automatic taxonomic literature analysis continues into the future.

## References

[1] Miller J, Dikow T, Agosti D, Sautter G, Catapano T, et al (2012) From taxonomic literature to cybertaxonomic content. BMC Biology 10: 87. 23114078 10.1186/1741-7007-10-87 PMC3485131

[2] Page RDM (2013) BioNames: Linking taxonomy, texts, and trees. PeerJ 1: e190 http://dx.doi.org/110.7717/peerj.7190. 24244913 10.7717/peerj.190 PMC3817598

[3] Kennedy JB, Kukla R, Paterson T (2005) Scientific Names Are Ambiguous as Identifiers for Biological Taxa: Their Context and Definition Are Required for Accurate Data Integration. In: Lüdascher B, Raschid L, editors. Data Integration in the Life Sciences: Proceedings of the Second International Workshop, San Diego, CA, USA, July 20–22 DILS 2005, LNBI: Springer. pp. 80–95.

[4] Franz NM, Cardona-Duque J (2013) Description of two new species and phylogenetic reassessment of Perelleschus Wibmer & O'Brien, 1986 (Coleoptera: Curculionidae), with a complete taxonomic concept history of Perelleschus sec. Franz & Cardona-Duque, 2013. Systematics and Biodiversity 11: 209–236.

[5] Hoffmann CK (1874) Crustacea. Leyden. 1–58 p.

[6] Kingsley JS (1880) Carcinological notes, No. II.-Revision of the Gelasimi. Proc Acad Nat Sci Phil 1880: 135–155.

[7] Nabout JC, Bini LM, Diniz-Filho JAF (2010) Global literature of fiddler crabs, genus *Uca* (Decapoda, Ocypodidae): Trends and future directions. Iheringia Ser Zool 100: 463–468.

[8] Rao KR, Fingerman M (1968) Dimorphic variants of the fiddler crab *Pca pugilator* and their chromatophore responses. Proc La Acad Sci 31: 27–39.

[9] Fielder DR, Rao KR, Fingerman M (1971) A female-limited lipoprotein and the diversity of hemocyanin components in the dimorphic variants of the fiddler crab, *Uca pugilator*, as revealed by disc electrophoresis. Comp Biochem Physiol 39B: 291–297.

[10] Novak A, Salmon M (1974) *Uca panacea*, a new species of fiddler crab from the Gulf coast of the United States. Proc Biol Soc Wash 87: 313–326.

[11] Vernberg FJ, Costlow JD Jr (1966) Studies on the physiological variation between tropical and temperate-zone fiddler crabs of the genus *Uca*. IV. Oxygen consumption of larvae and young crabs reared in the laboratory. Physiological Zoölogy 39: 36–52.

[12] Macnae W (1966) Mangroves in eastern and southern Australia. Aust J Bot 14: 67–104.

[13] Hess W (1865) Beiträge zur Kenntniss der Decapoden-Krebse ost-Australiens. Archiv für Naturgescicthe 31: 127–173.

[14] King DS, Siddall JB (1969) Conversion of α-ecdoysone to β-ecdysone by crustaceans and insects. Nature 221: 955–956. 5765507 10.1038/221955a0

[15] Crane J (1975) Fiddler Crabs of the World: Ocypodidae: Genus *Uca*. Princeton, NJ: Princeton University Press. 736 p.

[16] Gould AA (1841) A report on the invertebra of Massachusetts, comprising the Mollusca, Crustacea, Annelida, and Radiata. Cambridge, Massachusetts: Folsom, Wells, and Thurston. 373 p.

[17] George RW, Jones DS (1982) A revision of the fiddler crabs of Australia (Ocypodinae: *Uca*). Rec West Aust Mus Suppl 14: 1–99.

[18] Huber R, Klump J (2009) Charting taxonomic knowledge through ontologies and ranking algorithms. Computers and Geosciences 35: 862–868.

[19] Marcgrave G (1648) Historiæ Rerum Naturalium Brasiliæ. Historia Naturalis Brasiliae. Leyden and Amsterdam: Lugdun Batavorum et Amstelodami. pp. 1–293.

[20] Linnaeus C (1758) Systema Naturæ. 824 p.

[21] Rumphius GE (1705) D'Amboinsche rariteitkamer, behelzende eene beschryvinge van allerhande zoo weeke als harde schaalvisschen, te weeten raare krabben, kreeften, en diergelyke Zeedieren, als mede allerhande hoorntjes en schulpen, die men in d'Amboinsche Zee vindt: Daar beneven zommige mineraalen, gesteenten, en soorten van aarde, die in d'Amboinsche, en zommige omleggende Eilanden gevonden worden. Amsterdam: François Halma.

[22] Bott R (1973) Die Typus-Art der Gattung *Uca* Leach 1814 (Decapoda: Ocypodidae). Senckenb Biol 54: 311–314.

[23] Holthuis LB (1979) *Cancer vocans major* Herbst, 1782 (Crustacea, Decapoda): Request for the use of the plenary powers to validate a neotype. Z.N.(S). 2235. Bull Zool Nomen 35: 248–252.

[24] Tavares MS (1993) Toward the history of pre-Linnean carcinology in Brazil. In: Truesdale F, editor. Crustacean Issues 8: History of carcinology. Rotterdam: Balkema. pp. 7–29.

[25] Rathbun MJ (1897) A revision of the nomenclature of the Brachyura. Proc Biol Soc Wash 11: 153–167.

[26] Rathbun MJ (1915) New genera and species of American Brachyrhynchous crabs. Proc US Nat Mus 47: 117–129.

[27] Verwey J (1930) Einges über die Biologie Ost-Indischer Mangrovekrabben. Treubia 12: 167–261.

[28] Shih H-T, Kamrani E, Davie PJF, Liu M-Y (2009) Genetic evidence for the recognition of two fiddler crabs, *Uca iranica* and *U. albimana* (Crustacea: Brachyura: Ocypodidae), from the northwestern Indian Ocean, with notes on the *U. lactea* species-complex. Hydrobiologia 635: 373–382.

[29] Rosenberg MS (2013) The nomenclatural status of the two “spiny-wristed” fiddler crabs: *Uca spinicarpa* Rathbun, 1900, and *U. hesperiae* Crane, 1975 (Crustacea: Brachyura: Ocypodidae). Zootaxa 3716: 494–500. 26106787 10.11646/zootaxa.3716.3.10

